# Rapid growth in early childhood associated with young adult overweight and obesity – evidence from a community based cohort study

**DOI:** 10.1186/s41043-015-0012-2

**Published:** 2015-08-08

**Authors:** Ratneswary Sutharsan, Michael J. O’Callaghan, Gail Williams, Jake M. Najman, Abdullah A. Mamun

**Affiliations:** 1 School of Population Health, The University of Queensland, Brisbane, Australia; 2 Eskitis Institute, Griffith University, Brisbane, Australia; 3 Mater Children’s Hospital, Brisbane, and The University of Queensland, Brisbane, Australia; 4 School of Social Science, The University of Queensland, Brisbane, Australia

## Abstract

**Background:**

Rapid weight gain in early life may increase the risk of overweight and obesity in adulthood. We investigated the association between the rate of growth during early childhood and the development of overweight and obesity in young adults.

**Methods:**

We used a prospective cohort study of 2077 young adults who were born between 1981 and 1984 in Brisbane, Australia and had anthropometry measurements available at birth, 6 months, 5 years, 14 years and 21 years of age. The associations of rate of early growth with body mass index (BMI), waist circumference (WC) and waist-to-hip ratio (WHR) and their categories at 21 years were studied using multivariate analysis.

**Results:**

We found that rapid weight gain [> + 0.67 standard deviation score (SDS)] in the first 5 years of life was associated with young adults’ overweight status (BMI: adjusted OR = 2.35, 95 % CI, 1.82–3.03; WC: adjusted OR = 2.20, 95 % CI, 1.65–2.95). We also observed that slow weight gain in the first 5 years of age (< −0.67 SDS) was inversely associated with overweight (BMI: OR = 0.62, 95 % CI, 0.45–0.84). Such associations were not found with WHR. Rapid weight gain in the first 6 months of life increased the risk of overweight as defined by BMI (adjusted OR = 1.13, 95 % CI, 0.86–1.49) and WC (adjusted OR = 1.24, 95 % CI, 0.92–1.67), but these associations were not statistically significant.

**Conclusion:**

Rapid weight gain in the first 5 years of life in children increased their risk of a higher BMI and WC in young adulthood, in contrast slow weight gain was inversely associated with weight status at 21 years.

## Background

Globally, the prevalence of obesity has doubled during the last three decades, and in 2008, more than 10 % of the adult population was estimated to be obese [1]. There is insufficient evidence to consolidate prevention strategies targeted at adults or even to recognise the best age group for intervention. The potential risk of rapid postnatal weight gain on the development of obesity later in life has drawn considerable attention in the last decade. The association between rapid weight gain in infancy and obesity in later life has been reported in some observational studies [2–6], systematic reviews and meta-analyses [7–11]. A stronger association was found between postnatal weight gain and subsequent obesity in children compared to adults. Although some studies have tracked the longer term effects of early rapid weight gain [3, 4, 12–15], potential mid-childhood mediating factors were hardly taken into account. Information on prospectively tracking the effects of both slow and rapid early growth into adulthood obesity status as measured by body mass index (BMI), waist circumference (WC) and waist-to-hip (WHR), in the contemporary population with a larger sample is limited. None of the studies have examined whether or not slow growth protects against obesity.

The aim of this study is to investigate the association between early weight gain in children and their overweight or obesity status in young adulthood with consideration to mid-childhood factors. We hypothesise that rapid weight gain in early life increases the risk of overweight, slow weight gain protects against overweight and mid-childhood factors, including food, physical activity and television-viewing mediate the associations.

## Methods

The dataset used in this present study was collected from the Mater-University of Queensland of Pregnancy (MUSP), a prospective cohort study of 7223 women and their children who were born during the 1981–1984 period at the Mater Hospital in Brisbane, Australia. The Mater hospital is a tertiary hospital, located on the south side of the Brisbane River, which accommodated nearly 50 % of the deliveries in Brisbane during 1981–1983 [16]. The mothers who participated in this study were public healthcare patients comprising 58 % of the total mothers attending to the hospital. A detailed description of this cohort has been published previously [16, 17]. The mothers who gave birth to live singleton babies and did not give their babies for adoption constituted the MUSP birth cohort. The study has continued to measure factors indicative of the development, growth, health, learning and behaviour of offspring at critical stages of life: at the 6-month, 5-year, 14-year and 21-year follow-ups [16]. The average age of the young adults at 21 years follow-up was 20.65 years, with a range from 18.17 to 23.53 years. Children who were born at ≥37 weeks of gestation N = 1993 and consistently collected growth data at 6 month, 5 years, and 21 years were considered in this study.

Mothers consented to their participation and that of their babies. At 21 years, the young adults provided their consent to participate in the study. This study has been approved by the University of Queensland Ethics Committee and the Mater Hospital Ethics Committee.

In general, attrition rate was high in this long follow up study and individuals who were socially disadvantaged and had poor mental health were more likely to be lost of follow-up compared to respondents who remain in the study [16]. For this study, iformation was available for a sub-sample of 1993 offspring at birth, 5 years and 21 years. This attrition might have introduced some bias in the findings. However, distribution of important exposure and outcome variables included in the analyses did not vary considerably from the original cohort (Table 1).

**Table 1:** Comparison of mean values and the frequencies of crucial child and maternal characteristics from the main analysis group with the original MUSP cohort

		Original cohort		Main analysis set		
Parameter		N	% or Mean (SD)	N	% or Mean (SD)	*P* value*
Gender	Male	3579	51.7	1025	49.4	0.06
	Female	3348	48.3	1052	50.6	
Gestation (weeks)		6927	39.6 (1.27)	2077	39.6 (1.28)	1
Birth weight (g)		6926	3430 (469)	2077	3438 (462)	0.5
Z-score of birth weight		6925	0.05 (0.98)	2077	0.08 (0.96)	0.2
Z-score of weight at 6 months		5451	−0.19 (1.44)	2077	−0.17 (1.2)	0.6
Z-score of weight gain 0–6 months		5450	−0.25 (1.50)	2077	−0.25 (1.27)	1
Absolute weight at 5 years (kg)		3827	20.5 (3.1)	1910	20.5 (3.0)	1
Z-score weight at 5 years		3927	0.25 (1.01)	1910	0.28 (0.99)	0.7
Z-score of early childhood weight gain 0–5 years		3826	0.16 (1.19)	1910	0.14 (1.17)	1
Height at 21 years (cm)		2550	171.7 (9.2)	2077	172 (9.3)	0.3
Weight at 21 years (kg)		2522	71.7 (16.2)	2077	71.7 (16.2)	1
BMI at 21 years (kg/m^2^)		2522	24.2 (4.96)	2077	24.2 (4.9)	1
Waist circumference (cm)		2523	81.7 (12.4)	2074	81.7 (12.3)	1
Waist to hip ratio		2520	0.83 (0.08)	2072	0.83 (0.08)	1
Breast feeding	Yes ≥4 months	1911	29.9	731	35.3	<0.001
	Yes <4 months	3142	49.1	1000	48.2	
	Not at all	1344	21	342	16.5	
Parity–previous births	No birth	2804	40.5	863	41.6	0.1
	1	2128	30.7	650	31.3	
	2	1186	17.1	361	17.4	
	≥3	809	11.7	203	9.8	
Mother’s education–FVC	Incomplete high	1240	18.1	318	15.4	<0.001
	Complete high	4422	64.3	1322	64.1	
	Post high	1213	17.6	424	20.5	
Parents’ racial background	Caucasian	6008	89.4	1885	93.3	<0.001
	Asian	290	4.3	68	3.4	
	Abor-Islander	424	6.3	68	3.4	
Maternal smoking–FVC	None	4271	62.2	1373	66.6	0.001
	Smoker	2010	29.3	545	26.4	
	Heavy	581	8.5	143	6.9	
Mother’s age at birth (years)		6927	25.5 (5.10)	2077	26.0 (5. 0)	<0.001
Mother’s Pre-pregnancy BMI (kg/m^2^)		6395	21.9 (3.90)	1958	21.9 (3.7)	0.8

*BMI* body mass index, *FVC* first visit to clinic

*P-value indicates the level of significance; for categorical outcome, *X*2 test was applied; for continuous outcome, F test was used. Level of significance was set <0.05

### Measurements of outcome

At the 21-year follow-up, the weight and height of the participants were measured during physical assessments. Two measures of height and weight were taken and the average value was used. Measurements were taken on a scale (Wedderburn Personal Scales Model UWBW150, Wedderburn, Japan) accurate to 0.2 kg when the participants were lightly clothed. Height was measured with a portable stadiometer (Road Rod 214 Portable Stadiometer, USA) and BMI was computed from height and weight measurements [weight/height^2^ (kg/m^2^)]. BMI was categorised into normal weight (BMI <25 kg/m^2^), overweight (25–29.9 kg/m^2^) and obese (≥30 kg/m^2^) [18] groups.

Hip circumference (HC) and WC were also measured at the physical assessments as described previously [19]. The participants’ WHR was computed by dividing WC (cm) by HC (cm). WC was categorised into three groups [18, 19]: normal (females < 80 cm; males < 94 cm); overweight (females 80– < 88 cm; males 94–102 cm); and obese (females ≥88 cm; males ≥102 cm). WHRs were also categorised into three groups [18]: normal (females < 0.8; males < 0.9); overweight (females > 0.8– < 0.85; males > 0.9– < 1.0) and obese (females ≥ 0.85; males ≥ 1.0).

### Measurement of exposures

Postnatal weight gain: Birth weight was measured at the hospital and each child’s weight at 6 months was obtained by administrating 6-month follow-up questionnaires to the mothers. The children’s weight at birth and at 6 months was measured in grams. Information was obtained from mothers who were 4–9 months post-delivery (mean 6.07 months) and age and sex adjusted z-scores for weight were internally calculated. The z-score of the postnatal weight gain was computed by subtracting the z-score weight at birth from the z-score weight at the 6-month follow-up. The values of weight < −4SDS and > +5.0SDS were omitted in the analyses [8]. As previously considered by Ong et al. [2] the z-score of postnatal weight gain was categorised into slow (< −0.67SDS), gradual (≥ −0.67 to ≤ +0.67 SDS) and rapid growth (> + 0.67SDS).

Early childhood weight gain: At the 5-year follow-up, the weight of the participants was measured during their physical assessments. Measurements were taken on a scale accurate to 0.2 kg when the participants were lightly clothed. Two measures of weight were taken and the average value was used. The age- and sex-adjusted z-score values for weight at 5 years were computed. As shown previously, a z-score of weight gain greater than 0.67SDS was considered rapid growth from 0 to 5 years in children.

### Measurements of confounders and mediators

Potential confounding factors considered in the study include: gestation age (in weeks), parity, gender, maternal education, maternal pre-pregnancy BMI, parents’ racial background and maternal smoking. Maternal BMI (kg/m^2^) was computed from self-reported pre-pregnancy weight and height which were obtained during the first visit to the clinic (FVC). Weight measurements obtained at the first visit to clinic were highly correlated with self-reported maternal pre-pregnancy weight (r = 0.950). The socioeconomic status of the mothers was measured through a recommended proxy measure: maternal education, which was collected during the FVC of the study. Maternal smoking at FVC was also considered as confounding factors.

Breastfeeding reported at 6-month follow-up (never, <4 months 4 or more months) was considered a mediator. Adolescent’s pubertal development was considered a mediator. The development of puberty at 14 years was obtained through self-revealed information using Tanner drawings (Stages 1–5) [20, 21]. For statistical precision, these stages were further grouped into three categories: Stages 1 and 2; Stage 3; and Stages 4 and 5. Information on fast food consumption was obtained from self-administered questionnaires. Children’s physical activity was also measured from the self-administrated questionnaire in which they were asked about their engagement in exercise or sports in the previous week. The number of hours a child watched television on a weekday was obtained from their mothers’ reports.

### Statistical analysis

To determine any significant difference between the characteristics of the original cohort and the subsample included in the main analysis, *X*^2^ tests were performed for categorical variables (or for frequencies) and F-tests for continuously distributed variables. Multivariate logistic regression analyses were performed separately for each outcome variable after adjusting for potential confounders: gender, gestation, breast-feeding, parity, stages of puberty, fast food consumption at 14 years, television-viewing at 14 years, physical activity at 14 years, maternal education, maternal age at birth, maternal pre-pregnancy BMI, maternal smoking and race. As the exclusion of puberty did not influence the effect size, it was not included in the final model. In order to increase the statistical precision, the overweight and obese categories of the outcome variables were grouped together and considered as the overweight category.

The significant level was set as *P* < 0.05 (two-tailed). All the analyses were carried out using Stata IC Version 12.1 software (STATA Corp. Texas, USA).

## Results

The predictor and outcome measurements did not significantly differ between the original cohort and analytic sample (Table 1). Childhood factors in the main analysis set–physical activity, television-viewing and fast food consumption by family–did not differ significantly from those in the original cohort (results not shown). However, the children in the main analysis set were more likely to be breast fed (*P* = 0.001) than those in the original cohort. Compared to the original MUSP cohort, a significantly higher percentage of mothers with higher education and a Caucasian background and who were non-smokers (pre-pregnancy) were found in the main analysis set (*P* =0.001).

**Fig. 1:**
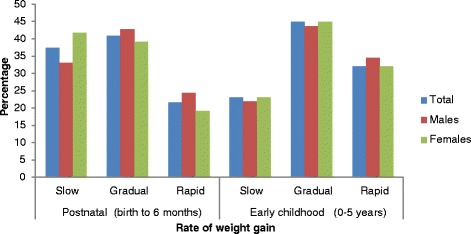
Rate of postnatal and early childhood weight gain (%) during postnatal and early childhood period in the MUSP cohort. [Slow: < −0.67 SDS; Gradual: ≥ −0.67– ≤ +0.67; Rapid: > + 0.67 SDS]

Nearly 22 % of infants showed rapid weight gain in their first six months of life. Male children showed a higher growth rate than female children (24.3 % and 19.1 %, respectively) (Fig. 1). Children who were first born, and bottle-fed and had less educated, smoking and younger mothers were more likely to grow rapidly in their first six months of life (results not shown). In the first five years of life, 32 % of children exhibited rapid growth whereas 23.1 % of children grew below 0.67SDS (Fig. 1). The rate of weight gain during the first five years of life was significantly associated with maternal education, smoking and parity (*P* < 0.001) (results not shown). When comparisons of important perinatal characteristics by rate of weight gain (slow, gradual and rapid) were made, it was found that rapid growers had a significantly higher gestation period and lower birth weight compared to children who exhibited gradual or slow growth.

Children who exhibited rapid weight gain had higher physical measurements at 21 years compared to gradual growers. Both males and females exhibited similar patterns. However, a significant difference was found between weight, BMI and WC measurements (*P* < 0.001) (Table 2).

**Table 2:** Distribution of outcome variables by the rate of early childhood weight gain (0–5 years) categories

Parameter	Rate of growth (0–5 years) by gender																			
	Males										Females									
	Slow (<0–0.67)			Gradual (≥ −0.67– ≤ +0.67)			Rapid (> + 0.67)			Gradual vs rapid	Slow (<0–0.67)			Gradual (≥ −0.67– ≤ +0.67)			Rapid (> + 0.67)			Gradual vs rapid
	N	Mean	SD	N	Mean	SD	N	Mean	SD	*P* value*	N	Mean	SD	N	Mean	SD	N	Mean	SD	*P* value*
Height at 21 years (cm)	206	176.3	7.02	411	178.8	6.5	325	179.7	6.71	0.07	236	164	6.4	446	165.2	5.91	286	166.6	6.05	0.002
Weight at 21 years (kg)	206	72.0	13.6	411	75.7	14.32	325	83.3	15.5	<0.001	236	60.3	12.5	446	65.6	14.0	286	72.8	17.5	<0.001
BMI at 21 years (kg/m^2^)	206	23.1	4.17	411	23.7	4.0	325	25.8	4.61	<0.001	236	22.4	4.38	446	24.0	5.06	286	26.2	6.13	<0.001
Waist circumference at 21 years (cm)	206	82.4	10.04	411	84.4	10.3	325	88.8	12.04	<0.001	235	74.7	10.35	445	77.7	11.5	285	82.3	13.75	<0.001
Waist to hip ratio (WHR) at 21 years	206	0.86	0.06	411	0.87	0.06	323	0.87	0.06	1.0	235	0.79	0.07	445	0.79	0.07	284	0.8	0.08	0.08

*P-value indicates the level of significance; F test was used. Level of significance was set <0.05

Based on their BMI measurements, the prevalence of overweight was 33.6 % among young adults, which was comparable to the national prevalence for this age interval during this period [22] and it did not significantly differ between the male and female groups (*P* = 0.7) (results not shown). When obesity status was classified by WC measurements, more females were overweight compared to males and the overall prevalence of overweight was in line with the classification of overweight by BMI. Based on WHR measurements, nearly 21 % of young adults were overweight and the prevalence was higher among males than females (25.6 % vs. 17.2 %, *P* < 0.001) (results not shown).

Table 3 shows the unadjusted and adjusted associations of the three outcome measures of overweight (BMI, WC and WHR) with early weight gain. Compared to gradual growth, rapid growth tended to increase the risk of overweight among young adults as measured by BMI, but the association was not statistically significant (adjusted OR = 1.13, 95 % CI, 0.86–1.49) . A similar trend was observed in the WC categories (adjusted OR = 1.24, 95 % CI, 0.92–1.67). The z-score of postnatal weight gain or the rate of postnatal growth did not have any impact on young adults’ WHR. Adjusting for confounders did not attenuate these associations.

**Table 3:** Association between early weight gain and young adults’ BMI, WC and WHR categories in the MUSP cohort: logistic regression analysis

	BMI categories^a^						WC categories^b^						WHR categories^c^					
	Unadjusted			Adjusted			Unadjusted			Adjusted			Unadjusted			Adjusted		
	OR	95 % CI		OR	95 % CI		OR	95 % CI		OR	95 % CI		OR	95 % CI		OR	95 % CI	
Z-score of postnatal weight gain	1.05	0.97	1.13	1.06	0.97	1.16	0.98	0.91	1.06	1.03	0.95	1.14	0.99	0.89	1.06	0.93	0.84	1.02
Rate of postnatal weight gain	Reference: Gradual (≥ −0.67 to ≤ +0.67)																	
Slow (< −0.67 SDS)	0.96	0.77	1.20	0.94	0.74	1.19	1.22	0.96	1.53	1.08	0.84	1.39	0.98	0.75	1.26	1.07	0.81	1.40
Rapid (> + 0.67 SDS)	1.13	0.87	1.47	1.13	0.86	1.49	1.20	0.91	1.59	1.24	0.92	1.67	1.05	0.77	1.42	0.97	0.71	1.32
Z-score of early childhood weight gain (0–5 years)	1.62	1.46	1.79	1.70	1.51	1.90	1.38	1.24	1.54	1.49	1.31	1.68	1.14	1.01	1.27	1.10	0.98	1.24
Rate of early childhood weight gain (0–5 years)	Reference: Gradual (≥ −0.67 to ≤ +0.67)																	
Slow (< −0.67 SDS)	0.69	0.51	0.92	0.62	0.45	0.84	0.89	0.63	1.24	0.78	0.55	1.11	0.75	0.52	1.07	0.75	0.52	1.08
Rapid (> + 0.67 SDS)	2.31	1.83	2.93	2.35	1.82	3.03	1.94	1.49	2.54	2.20	1.65	2.95	1.25	0.94	1.66	1.16	0.87	1.57

Variables considered in the adjusted model: gender, gestation, breast-feeding, parity, fast food consumption at 14 years, television-viewing at 14 years, physical activity at 14 years, maternal education, maternal age at birth, maternal pre-pregnancy BMI, maternal smoking and race

*OR* odds ratio, *SDS* standard deviation score, *CI* confidence interval, *BMI* body mass index, *WC* waist circumference, *WHR* waist-to-hip ratio

^a^Adjusted analysis: 0–6 months, *N* = 1768; 0–5 years, *N* = 1601

^b^Adjusted analysis: 0–6 months, *N* = 1769; 0–5 years, *N* = 1396

^c^Adjusted analysis: 0–6 months, *N* = 1768; 0–5 years, *N* = 1395

While slow growth reduced the risk of developing overweight/obesity (classified by BMI) in young adulthood (adjusted OR = 0.62, 95 % CI, 0.45–0.84), rapid growth substantially increased that risk compared to gradual growers (adjusted OR = 2.35, 95 % CI, 1.82–3.03) (Table 3). Rapid weight gain in the first five years of life increased the risk of becoming overweight more than twofold. A similar trend was observed for WC categories. When overweight and obesity was defined by WC, the children who gained weight rapidly in their first five years of life had over a two-fold increased risk of developing overweight in young adulthood (adjusted OR = 2.20, 95 % CI,1.65–2.95). Unlike BMI, slow growth was not strongly associated with WC status. Although rapid growth increased the risk of overweight status measured by WHR, the association was not significant (unadjusted OR = 1.25, 95 % CI, 0.94–1.66; adjusted OR = 1.16, 95 % CI, 0.87–1.57) (Table 3).

## Discussion

Using a large community-based cohort study, we found that the rate of weight gain in the first six months of life was not significantly associated with BMI, WC or WHR in young adults. When excess weight gain occurred in children over a more extended period of time (birth to 5 years), it increased their risk of presenting with a higher BMI and WC in young adulthood. Slow growth appeared to reduce the risk of developing overweight, but the association was only significant with BMI. Adult overweight status measured by WHR was not significantly influenced by early weight gain. All these associations were not explained by the potential confounding and mediating factors.

A stronger association between postnatal weight gain and a subsequent higher BMI can be found in childhood [2, 5, 6, 23–29] and adolescence [13, 30, 31]. Many studies that reported a strong positive association between rapid weight gain and obesity later in life measured exposure and outcome at closer time-points [5, 26–28, 32]. From the MUSP cohort, Mamun et al. [33] reported a positive association between postnatal weight gain (1 g/day in the first six months of life) and overweight at 5 and 14 years of age. In a recent study, any significant association between weight gain during 0–1 years or 1–2.5 years and obesity in young adults was not found [14].. McCarthy et al. [15] conducted detailed analyses from anthropometric measures at 14 time-points from birth to 5 years and then at 25 years of age, and found a positive association between infant growth and BMI at 25 years. However, weight gain between 21 months and 5 years (β = 0.99, *P* < 0.001) predicted a stronger association than between 0 and 5 months (β = 0.43, *P* = 0.02). Although the effect size of rapid postnatal growth from this current study was comparable to the findings of McCarthy et al. [15], it was not statistically significant (adjusted β = 0.45, SE = 0.30, *P* = 0.1). Whitaker et al. [34] also reported that obesity before 3 years of age was weakly correlated with adult obesity. It is likely that many mediating factors in adolescence could have exerted an influence on the association and attenuated the effect at 21 years. However, our results are in contrast to some previous studies that had sample sizes <700 [3, 4, 13]. In industrialised countries, overweight is believed to be associated with poor socioeconomic factors [35]. In the MUSP cohort, a higher percentage of mothers with low socio economic status were reported to drop out in the follow-ups [16], and this was also observed in the main analysis set. Therefore, this bias could have attenuated the effect size of the association between rapid early weight gain and overweight in young adulthood.

Nutrition and life-style factors during early childhood could have a greater impact on developing the overweight phenotype than that during postnatal period. In a recent randomised control trial, the effectiveness of early intervention on healthy food intake and physical activity among Australian infants was studied [36]. The authors reported that the intervention group had a significantly lower BMI at 2 years of age than the control group (0.29 kg/m^2^, 95 % CI, −0.55 to −0.02). They found measurable differences between the groups in infants’ vegetable consumption, food for reward and television-viewing [36]. These results suggest that early modifiable infant factors contribute considerably towards the development of overweight and obesity among children.

WC and WHR are better measurements of abdominal obesity, and the fat mass index is also strongly correlated with WC [31] and in the present study, rapid weight gain during 0–6months increased young adults’ WC, but the association was not significant. However this effect size is comparable with a previous study (N = 248) where weight gain 0–6 months resulted in an increased WC at 17 years (adjusted β = 1.40, *P* < 0.004) [13]. Similar results were reported for the effects of rapid gain in the first three months of life in young adults [37]. It was not possible in this study to ascertain whether the observed increase in WC was due to higher symmetrical growth or abdominal obesity; however, the effects of early rapid weight gain were not substantially attenuated when adjusted for critical factors such as race, maternal pre-pregnancy BMI, physical activity and puberty. Although rapid growth at both time points measured in this study appeared to increase the risk of a higher WHR, it was not statistically significant. Similar findings were observed by McCarthy et al. [15] who found that weight gain at different intervals (0–5months, 5–21months and 21 months–5 years) failed to predict any significant changes in WHR at 25 years of age. WHR is an indirect measure of metabolic disorders. As a result, it is also possible that young adulthood is still too early to differentially express the adverse effects of rapid growth through WHR although it could well be expressed in late young adulthood or mid-adulthood. Most studies reporting an association between postnatal weight gain and subsequent obesity have considered potential perinatal confounders but rarely childhood factors, which were considered in multivariate analysis. However, the validity of self-reported physical activity and television viewing may be suboptimum. Intake of fat food was measured through the frequency of fast food consumption by the family, and it may not be an accurate measure of fat intake by individuals.

Longer-term follow-up of the participants was one of the strength of the study. Measurements of weight and height by trained persons, such as those used in MUSP are considered to have a higher accuracy [35].

However, there are some limitations in the study as well. The MUSP cohort predominantly consists of a middle- to-lower socioeconomic families and attrition was considerably high [16]. However, our analysis and previous analyses samples from MUSP cohort show a smaller impact on findings due to attrition [38]. Postnatal weight was reported by mothers during the 6-month follow-up, thus limiting the validity of the measurement. It was also not possible to define the postnatal period with regard to weight gain as the mothers were contacted 4–9 months post-delivery (mean 6.07 months). These systematic errors could have biased the association and the ability to predict the critical postnatal period. There were concerns about the accuracy of the WC measurements during the 21-year follow-up of the study [19]. As the analyses employed here compared the mean values among groups and not the absolute values, it is unlikely that the observed association was substantially affected.

## Conclusions

The findings of this prospective cohort study confirm that an increased rate of weight gain in the first five years of life is associated with a greater risk of overweight and obesity, as defined by both BMI and WC, in young adulthood. In contrast, slow early childhood growth was associated with lower young adulthood BMI levels. Waist-to-hip ratios at 21 years did not predict the adverse effects of early weight gain which could become more evident in middle age. The findings of this study suggest that slow or optimal growth in early life, especially in first 5 years of life, may protect or reduce the young adult overweight and obesity.

## References

[1] WHO. Obesity and overweight. World Health Organisation. Fact Sheet No 311 2011 04 Aug 2013. Available from: http://www.who.int/mediacentre/factsheets/fs311/en/index.html.

[2] Ong KKL, Ahmed ML, Emmett PM, Preece MA, Dunger DB (2000). Association between postnatal catch-up growth and obesity in childhood: prospective cohort study. Br Med J.

[3] Stettler N, Stallings VA, Troxel AB, Zhao J, Schinnar R, Nelson SE (2005). Weight gain in the first week of life and overweight in adulthood–A cohort study of European American subjects fed infant formula. Circulation.

[4] Stettler N, Kumanyika SK, Katz SH, Zemel BS, Stallings VA (2003). Rapid weight gain during infancy and obesity in young adulthood in a cohort of African Americans. Am J Clin Nutr.

[5] Stettler N, Zemel BS, Kumanyika S, Stallings VA (2002). Infant weight gain and childhood overweight status in a multicenter, cohort study. Pediatrics.

[6] Tanaka T, Matsuzaki A, Kuromaru R, Kinukawa N, Nose Y, Matsumoto T (2001). Association between birthweight and body mass index at 3 years of age. Pediatr Int.

[7] Baird J, Fisher D, Lucas P, Kleijnen J, Roberts H, Law C (2005). Being big or growing fast: systematic review of size and growth in infancy and later obesity. BMJ.

[8] Druet C, Stettler N, Sharp S, Simmons RK, Cooper C, Davey Smith G (2012). Prediction of childhood obesity by infancy weight gain: an individual-level meta-analysis. Paediatr Perinat Epidemiol.

[9] Monteiro PO, Victora CG (2005). Rapid growth in infancy and childhood and obesity in later life[mdash]a systematic review. Obes Rev.

[10] Ong K, Loos R (2006). Rapid infancy weight gain and subsequent obesity: systematic reviews and hopeful suggestions. Acta Paediatr.

[11] Monasta L, Batty GD, Cattaneo A, Lutje V, Ronfani L, Van Lenthe FJ (2010). Early-life determinants of overweight and obesity: a review of systematic reviews. Obes Rev.

[12] Demerath EW, Reed D, Choh AC, Soloway L, Lee M, Czerwinski SA (2009). Rapid Postnatal Weight Gain and Visceral Adiposity in Adulthood: The Fels Longitudinal Study. Obesity.

[13] Ekelund U, Ong K, Linné Y, Neovius M, Brage S, Dunger DB (2006). Upward weight percentile crossing in infancy and early childhood independently predicts fat mass in young adults: the Stockholm Weight Development Study (SWEDES). Am J Clin Nutr.

[14] Fåhraeus C, Wendt L-K, Nilsson M, Isaksson H, Alm A, Andersson-Gäre B (2012). Overweight and obesity in twenty-year-old Swedes in relation to birthweight and weight development during childhood. Acta Paediatr.

[15] McCarthy A, Hughes R, Tilling K, Davies D, Davey Smith G, Ben-Shlomo Y (2007). Birth weight; postnatal, infant, and childhood growth; and obesity in young adulthood: evidence from the Barry Caerphilly Growth Study. Am J Clin Nutr.

[16] Najman J, Bor W, O’Callaghan M, Williams G, Aird R, Shuttlewood G (2005). Cohort Profile: The Mater-University of Queensland Study of Pregnancy (MUSP). Int J Epidemiol.

[17] Keeping JD, Najman JM, Morrison J, Western JS, Andersen MJ, Williams GM (1989). A prospective longitudinal study of social, psychological and obstetric factors in pregnancy: response rates and demographic characteristics of the 8556 respondents. BJOG.

[18] WHO. Obesity: preventing and managing the global epidemic. Report of a WHO consultation. World Health Organisation Tech Rep Ser 2000; 894: 1–253. 2000 22/03/2012. Available from: http://whqlibdoc.who.int/trs/who_trs_894.pdf. 11234459

[19] Liddle K, O’Callaghan M, Mamun A, Najman J, Williams G (2012). Comparison of body mass index and triceps skinfold at 5 years and young adult body mass index, waist circumference and blood pressure. J Paediatr Child Health.

[20] Tanner J (1962). Growth at Adolescence: Blackwell: Oxford.

[21] Mamun A, Hayatbakhsh M, O’Callaghan M, Williams G, Najman J (2009). Early overweight and pubertal maturationFpathways of association with young adults’ overweight: a longitudinal study. Int J Obes.

[22] Magarey AM, Daniels LA, Boulton TJ (2001). Prevalence of overweight and obesity in Australian children and adolescents: reassessment of 1985 and 1995 data against new standard international definitions. Med J Aust.

[23] Stettler N, Bovet P, Shamlaye H, Zemel BS, Stallings VA, Paccaud F (2002). Prevalence and risk factors for overweight and obesity in children from Seychelles, a country in rapid transition: the importance of early growth. Int J Obes.

[24] Karaolis-Danckert N, Buyken AE, Bolzenius K, de Perim Faria C, Lentze MJ, Kroke A (2006). Rapid growth among term children whose birth weight was appropriate for gestational age has a longer lasting effect on body fat percentage than on body mass index. Am J Clin Nutr.

[25] Kinra S, Baumer JH, Davey Smith G (2005). Early growth and childhood obesity: a historical cohort study. Arch Dis Child.

[26] Taveras EM, Rifas-Shiman SL, Sherry B, Oken E, Haines J, Kleinman K (2011). Crossing Growth Percentiles in Infancy and Risk of Obesity in Childhood. Arch Pediatr Adolesc Med.

[27] Taveras EM, Rifas-Shiman SL, Belfort MB, Kleinman KP, Oken E, Gillman MW (2009). Weight Status in the First 6 Months of Life and Obesity at 3 Years of Age. Pediatrics.

[28] Terry MB, Wei Y, Esserman D, McKeague IW, Susser E (2011). Pre- and postnatal determinants of childhood body size: cohort and sibling analyses. J Dev Origins Health Dis.

[29] Durmuş B, Kruithof CJ, Gillman MH, Willemsen SP, Hofman A, Raat H (2011). Parental smoking during pregnancy, early growth, and risk of obesity in preschool children: the Generation R Study. Am J Clin Nutr.

[30] Monteiro POA, Victora CG, Barros FC, Monteiro LMA (2003). Birth size, early childhood growth, and adolescent obesity in a Brazilian birth cohort. Int J Obes.

[31] Eriksson M, Tynelius P, Rasmussen F (2008). Associations of birthweight and infant growth with body composition at age 15–the COMPASS study. Paediatr Perinat Epidemiol.

[32] Parsons TJ, Power C, Manor O (2001). Fetal and early life growth and body mass index from birth to early adulthood in 1958 British cohort: longitudinal study. BMJ.

[33] Mamun AA, Lawlor DA, O’Callaghan MJ, Williams GM, Najman JM (2005). Family and early life factors associated with changes in overweight status between ages 5 and 14 years: findings from the Mater University Study of Pregnancy and its outcomes. Int J Obes (Lond).

[34] Whitaker RC, Wright JA, Pepe MS, Seidel KD, Dietz WH (1997). Predicting obesity in young adulthood from childhood and parental obesity. N Engl J Med.

[35] Lobstein T, Baur L, Uauy R (2004). Obesity in children and young people: a crisis in public health. Obesity reivews.

[36] Wen L, Baur L, Simpson J, Rissel C, Wardle K, Flood V (2012). Effectiveness of home based early intervention on children’s BMI at age 2: randomised controlled trial. BMJ.

[37] Leunissen RWJ, Kerkhof GF, Stijnen T, Hokken-Koelega A (2009). Timing and Tempo of First-Year Rapid Growth in Relation to Cardiovascular and Metabolic Risk Profile in Early Adulthood. JAMA.

[38] Mamun AA, O’Callaghan MJ, Williams GM, Najman JM (2013). Change in maternal body mass index is associated with offspring body mass index: a 21-year prospective study. Eur J Nutr.

